# Immunization of Nile Tilapia (*Oreochromis niloticus*) Broodstock with Tilapia Lake Virus (TiLV) Inactivated Vaccines Elicits Protective Antibody and Passive Maternal Antibody Transfer

**DOI:** 10.3390/vaccines10020167

**Published:** 2022-01-21

**Authors:** Thao Thu Mai, Pattanapon Kayansamruaj, Chayanit Soontara, Pattarawit Kerddee, Dinh-Hung Nguyen, Saengchan Senapin, Janina Z. Costa, Jorge del-Pozo, Kim D. Thompson, Channarong Rodkhum, Ha Thanh Dong

**Affiliations:** 1 Center of Excellence in Fish Infectious Diseases (CE FID), Department of Veterinary Microbiology, Faculty of Veterinary Science, Chulalongkorn University, Bangkok 10330, Thailand; thaomt.hcmbiotech@gmail.com (T.T.M.); henrynguyen.vnua@gmail.com (D.-H.N.); 2 The International Graduate Program of Veterinary Science and Technology (VST), Faculty of Veterinary Science, Chulalongkorn University, Bangkok 10330, Thailand; 3 Division of Aquacultural Biotechnology, Biotechnology Center of Ho Chi Minh City, Ho Chi Minh 700000, Vietnam; 4 Center of Excellence in Aquatic Animal Health Management, Faculty of Fisheries, Kasetsart University, Bangkok 10900, Thailand; pattanapon.k@ku.th (P.K.); chayanit.soo@ku.th (C.S.); Pattarawit.ker@ku.th (P.K.); 5 Fish Health Platform, Center of Excellence for Shrimp Molecular Biology and Biotechnology (Centex Shrimp), Faculty of Science, Mahidol University, Bangkok 10400, Thailand; saengchan@biotech.or.th; 6 National Center for Genetic Engineering and Biotechnology (BIOTEC), National Science and Technology Development Agency (NSTDA), Khlong Nueng 12120, Thailand; 7 Aquaculture Research Group, Moredun Research Institute, Edinburgh EH26 0PZ, UK; janina.costa@moredun.ac.uk (J.Z.C.); kim.thompson@moredun.ac.uk (K.D.T.); 8 Infection and Immunity Division, Roslin Institute, Edinburgh EH25 9RG, UK; Jorge.Del.Pozo@ed.ac.uk; 9 Aquaculture and Aquatic Resources Program, Department of Food, Agriculture and Bioresources, School of Environment, Resources and Development, Asian Institute of Technology, Khlong Nueng 12120, Thailand

**Keywords:** tilapia broodstock, inactivated vaccines, maternal passive immunity, antibody

## Abstract

Tilapia lake virus (TiLV), a major pathogen of farmed tilapia, is known to be vertically transmitted. Here, we hypothesize that Nile tilapia (*Oreochromis niloticus*) broodstock immunized with a TiLV inactivated vaccine can mount a protective antibody response and passively transfer maternal antibodies to their fertilized eggs and larvae. To test this hypothesis, three groups of tilapia broodstock, each containing four males and eight females, were immunized with either a heat-killed TiLV vaccine (HKV), a formalin-killed TiLV vaccine (FKV) (both administered at 3.6 × 10^6^ TCID_50_ per fish), or with L15 medium. Booster vaccination with the same vaccines was given 3 weeks later, and mating took place 1 week thereafter. Broodstock blood sera, fertilized eggs and larvae were collected from 6–14 weeks post-primary vaccination for measurement of TiLV-specific antibody (anti-TiLV IgM) levels. In parallel, passive immunization using sera from the immunized female broodstock was administered to naïve tilapia juveniles to assess if antibodies induced in immunized broodstock were protective. The results showed that anti-TiLV IgM was produced in the majority of both male and female broodstock vaccinated with either the HKV or FKV and that these antibodies could be detected in the fertilized eggs and larvae from vaccinated broodstock. Higher levels of maternal antibody were observed in fertilized eggs from broodstock vaccinated with HKV than those vaccinated with FKV. Low levels of TiLV-IgM were detected in some of the 1–3 day old larvae but were undetectable in 7–14 day old larvae from the vaccinated broodstock, indicating a short persistence of TiLV-IgM in larvae. Moreover, passive immunization proved that antibodies elicited by TiLV vaccination were able to confer 85% to 90% protection against TiLV challenge in naïve juvenile tilapia. In conclusion, immunization of tilapia broodstock with TiLV vaccines could be a potential strategy for the prevention of TiLV in tilapia fertilized eggs and larvae, with HKV appearing to be more promising than FKV for maternal vaccination.

## 1. Introduction

Tilapia have become one of the most important freshwater fish species to be farmed globally, and are now farmed in over 140 countries, with global production expected to reach 7.3 million tons in 2021 [1,2]. Intensification of farming systems tends to lead to poor water quality and increased risk of infectious disease outbreaks [3]. Tilapia lake virus (TiLV), also known as *Tilapia tilapinevirus*, is one of the most significant infectious agents causing relatively high mortality and economic losses for tilapia farmers [4]. It is a single-stranded RNA virus with ten genomic segments and ranging from 55 to 100 nm in diameter [5,6,7,8]. The mortality rate in natural TiLV outbreaks ranges from 20% to 90% [5,6,9,10], while cumulative mortalities from experimental infection range from 66% to 100% [11,12]. The virus can infect fertilized eggs, yolk-sac fish, fry, fingerlings and adult fish [13,14]. Recent studies have reported that TiLV can also be transmitted vertically from infected broodstock to their offspring [15,16].

Vaccination is an effective strategy to prevent infectious diseases in aquaculture. Several vaccines have been described for the control of TiLV in tilapia. Attenuated TiLV strains induced by 17 and 20 consecutive passages on a permissive cell line showed considerable protection after intraperitoneal injection, yielding a relative percent survival (RPS) of 56–58% [17]. Another study reported that β-propiolactone-inactivated vaccines in combination with adjuvant Montanide IMS 1312 VG for intraperitoneal injection resulted in RPS values ranging from 32.1% to 85.7%, depending on the dose used [18]. A DNA vaccine, consisting of a vector pVAX1 containing the gene encoding segment 8 (VP20), was used for primary vaccination, followed by a booster vaccination with a recombinant VP20 (rVP20) in adjuvant M402. This vaccine combination gave 72.5% protection in vaccinated fish, which was higher than the level obtained with the DNA or the VP20 vaccines alone [19]. Most recently, water-based inactivated vaccines prepared with heat-killed or formalin-killed TiLV were shown to provide good levels of protection in juvenile tilapia, with RPS of 71.3% and 79.6%, respectively [20]. It has also been reported that TiLV vaccines can induce both humoral immunity and cell mediated immunity [18,19,20].

Active immunity is defined as protection against a pathogen following exposure to the pathogen or pathogen-derived antigens [21]. Vaccination is one of the mechanisms used to develop active immunity in fish. Protection is provided through both humoral immunity and cell-mediated immunity. In addition, an immunological memory is produced after vaccination, conferring protection when the fish is subsequently exposed to the same pathogen at a later date [22]. Conversely, passive immunity usually refers to the transfer of antibodies from one individual to another to provide protection against an infectious agent [23]. It is thought that passive immunity in fish can be acquired through the transfer of protective antibodies from vaccinated broodstock to their offspring [24]. Of the immunoglobulin classes present in fish, IgM has been reported to be maternally transferred into the immature oocytes during the vitellogenin formation process and later absorbed throughout the egg in the follicular cells [25,26,27].

Maternal antibody transfer through vaccination of broodstock has been applied in several fish species to reduce the risk of vertical disease transmission and increase larval survival. For example, a significant reduction in vertical transmission of nervous necrosis virus (NNV) was observed in the group (*Epinephelus coioides*) vaccinated with inactivated NNV. The virus was detected in the eggs of unvaccinated broodstock, but not in the eggs of vaccinated fish [27]. In another study, when tilapia broodstock were vaccinated with an inactivated *Streptococcus agalactiae* vaccine, higher larval survival was observed compared to larvae from unvaccinated broodstock [28].

In the current study, we investigate levels of TiLV-specific antibodies in Nile tilapia broodstock after immunization with either heat-killed vaccine (HKV) or formalin-killed vaccine (FKV), and the role of these antibodies in protecting juvenile tilapia against TiLV through passive immunization. In parallel, we also assess the transfer of maternal antibodies from vaccinated broodstock to their fertilized eggs and larvae.

## 2. Materials and Methods

### 2.1. Experimental Fish

Thirty-six Nile tilapia broodstock (12 males and 24 females, body weight 600–800 g) were kindly provided by the Fisheries Research Station, Faculty of Fisheries, Kasetsart University, Thailand, which were clinical healthy, sexually mature and ready for breeding. These fish were originally obtained from a hatchery with no previous history of TiLV infection. Fish were separated by gender and acclimated in two 3000-L plastic tanks with aeration. The fish were maintained in an indoor system at a water temperature of 29 ± 1 °C and fed twice daily at 3% of their body weight with a commercial pellet. Fish were cultured in dechlorinated tap water and half of the water volume was renewed weekly. Prior to the experiment, blood samples were taken from ten randomly selected tilapia and tested for the presence of TiLV using RT -qPCR [29], and their TiLV-free status at the point of sampling was confirmed. All the animal experiments and procedures used in this study were ethically approved by the Kasetsart University Institutional Animal Care and Use Committee (ACKU62-FIS-008).

### 2.2. Vaccine Preparation

The TiLV inactivated vaccines were prepared as described previously by Mai et al. (2021). Briefly, TiLV strain TH-2018-K, which was isolated from Nile tilapia during a TiLV outbreak in Thailand in 2018 [20], was propagated on E11 cells, in Leibovitz’s L15 medium (Sigma, Saint Louis, MO, USA) containing 5% fetal bovine serum, until a cytopathic effect (CPE) of approximately 80% of the cell monolayer was achieved. The supernatant containing the virus was collected and clarified to remove cell debris, by centrifuging at 4500× *g* for 5 min at 4 °C. Virus concentration was determined using 50% tissue culture infectious dose (TCID_50_/mL) [30]. The virus was inactivated by either heating at 60 °C for 2.5 h or incubating in a 0.006% formalin solution (16.2 µL formalin 0.37% in 1X phosphate-buffered saline [1× PBS, 137 mM NaCl, 2.7 mM KCl, 10 mM Na_2_HPO_4_ and 1.8 mM KH_2_PO_4_, pH 7.4] per 1.0 mL viral stock) at 25 °C for 24 h. Viral infectivity was tested on E11 cells and successful inactivation was confirmed when no CPE was observed after 7 days. The vaccines were stored at 4 °C until used. The virus concentration was determined to be 1.8 × 10^7^ TCID_50_ per mL before being used. All chemicals used were purchased from Merck (Kenilworth, NJ, USA).

### 2.3. Immunization, Breeding and Sampling

**Figure 1:**
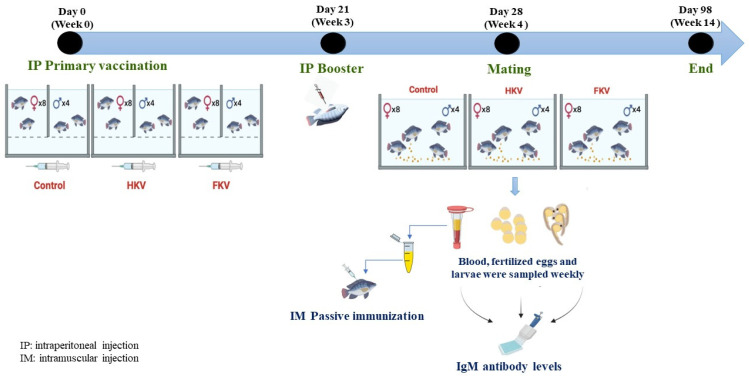
Diagram illustrating the experimental design for broodstock TiLV vaccination, mating and sampling.

The experimental design for broodstock vaccination is shown in Figure 1. Three groups of broodstock were divided into three tanks, each containing four males and eight females, as previously described [31], where gender groups were separated by a partition. Before vaccination and blood sampling, fish were anaesthetized using clove oil (100 ppm). Fish were immunized by interperioneal (IP) injection with 0.2 mL of either HKV, FKV (3.6 × 10^6^ TCID_50_ per fish) or L-15 medium (control), respectively. Three weeks after the primary vaccination, a booster was administered in the same manner. All fish were clinically healthy after receiving the vaccines. One week after the booster vaccination, the tank partitions were removed and male and female broodstock were allowed to mix and mate. In each group, blood (~500 µL per fish) was collected from one male and one or two female broodstock that did not have fertilized eggs in their mouth weekly after mating from the caudal vessel using a 25G needle. Blood was allowed to clot and sera were collected by centrifuging the blood samples at 4000× *g* for 15 min. Sera were stored at −20 °C for analysis.

### 2.4. Egg and Larvae Collection

Fertilized eggs were collected 6 to 14 weeks post primary vaccination (wppv). Approximately 50 fertilized eggs (constituting a batch of fertilized eggs) were collected weekly from mouths of female broodstock that retained eggs in their mouth. Each batch of eggs was kept in a 1.5 mL tube at −20 °C to test by ELISA. The remaining eggs were placed in a conical incubation tank where they were continually circulated and thoroughly oxygenated. When eggs began to hatch, the water flow was reduced, allowing the hatched larvae to swim to the surface of the tank, open their mouth and engulf air. The hatched larvae were then transferred to rearing trays where they were allowed to swim freely. Approximately 50 larvae (forming a batch of larvae) were collected between 1 and 14 days post-hatching (dph) and stored at −20 °C for ELISA analysis.

After mating, not all female broodstock in the three groups produced eggs simultaneously for egg sampling. Fertilized eggs were obtained 6, 7, 9, 10, 12-wppv from the control group; 7, 8, 9, 11, 12 and 14-wppv from the HKV group and 6, 7, 10, 11, 12, 13, 14-wppv from the FKV group. The number of batches of fertilized eggs and larvae that were collected from each batch of hatched eggs at different time points are indicated in Table 1.

### 2.5. Measurement of Anti-TiLV IgM Antibody Levels by ELISA

Anti-TiLV IgM antibody levels were measured in broodstock sera, fertilized eggs and larvae by ELISA. A sample of 100 mg eggs or larvae was homogenized on ice in 400 µL PBS 1X containing 0.05% Tween 20 (BioRad, Berkeley, CA, USA). The samples were then centrifuged at 5000× *g* for 10 min at 4 °C and the supernatant collected. Ninety-six well polystyrene ELISA plates (Corning, Shanghai, China) were coated with 100 µL of 0.01% poly-L-lysine solution (Sigma, Saint Louis, MO, USA) for 1 h at 28 °C. They were rinsed three times with low salt wash buffer (LSWB, 2 mM Tris; 38 mM NaCl; 0.005% Tween 20, pH 7.3) before incubating them with 100 µL of either heat- or formalin-killed TiLV (1.8 × 10^7^ TCID_50_ per mL) overnight at 4 °C. The following day, 50 µL glutaraldehyde 0.05% (EMS, USA) was added and incubated for 20 min at 28 °C, then wells were rinsed three times with LSWB. Non-specific binding sites were blocked by the addition of 100 µL of 1% bovine serum albumin (BSA, Sigma, Saint Louis, MO, USA) in 1× PBS for 2 h at 28 °C. During this time 100 µL of either fish sera (diluted 1:1024 in 1× PBS), egg supernatant (diluted 1:8 in 1× PBS) or larvae supernatant (diluted 1:2 in 1× PBS) were prepared. The blocking reagent was removed from the wells and the diluted samples added to the ELISA plate, which was subsequently incubated overnight at 4 °C. The plates were rinsed five times with high salt wash buffer (HSWB, 2 mM Tris; 50 mM NaCl; 0.01% Tween 20, pH 7.7) before incubating them with an anti-tilapia IgM monoclonal antibody [32] (diluted 1:200 in 1× PBS + 1% BSA) for 2 h at 28 °C. The plates were then rinsed five times with HSWB, followed by incubation with goat anti-mouse antibody conjugated with horseradish peroxidase (diluted 1:3000 in LSWB + 1% BSA) for 1 h at 28 °C. Finally, the plates were rinsed five times with HSWB, each well was filled with 100 µL of 3,3′,5,5′-Tetramethylbenzidine (TMB), and the reaction was allowed to develop in the dark for 5–10 min before adding 50 µL of stop solution (2 M H_2_SO_4_). Optical density was measured at a wavelength of 450 nm using the microplate reader (SpectraMax ID3, San Jose, CA, USA). The OD_450_ cut-off value was calculated as standard deviation (SD) × f + mean OD_450_ value of negative control wells, where the f values were the standard deviation multipliers corresponding to the 95% confidence levels at sample sizes of 2–30 [33]. Negative controls were the fish sera, fertilized eggs or larval supernatant from the control group. The cut-off OD_450_ values for broodstock sera, fertilized eggs and larval supernatant were calculated as indicated in Table 2. Unless specifically mentioned in the text, all chemicals used were purchased from Merck (Kenilworth, NJ, USA).

### 2.6. Passive Immunization

Sera pooled from three female broodstock in each group, including HKV with OD_450_ values of 0.911, 1.007 and 0.647, FKV with OD_450_ values of 1.048, 0.889 and 0.944 and the control group with OD_450_ values of 0.057, 0.058 and 0.060 (1:1024 dilution in 1× PBS before pool) were used for the passive immunization experiment. Clinically healthy tilapia juveniles (body weight 20.3 ± 6.7 g; length 10.9 ± 0.7 cm) were acclimated in dechlorinated tap water using 100-L tanks, with a density of 20 fish per tank. Prior to the experiment, five fish were randomly tested for the presence of TiLV using a RT-qPCR assay [29] and confirmed as negative. Prior to immunization, fish were anaesthetized using clove oil (100 ppm). Three groups of 20 fish were immunized intramuscularly (IM) in the dorsal musculature with pooling sera (50 µL/fish) from HKV (group 1), FKV (group 2) and control (group 3). Another group of 20 fish (group 4) were IM immunized with L15 as negative control. Twenty-four hours after passive immunization, groups 1, 2 and 3 were IP challenged with TiLV TH-2018 (9 × 10^5^ TCID_50_ per fish) and group 4 were challenged with L15 without virus. Cumulative mortalities were recorded for 21 days. Relative percent survival (RPS) was calculated as follows:

(1) $$\mathrm{RPS}=\left(1-\frac{\%\;\mathrm{cumulative}\;\mathrm{mortality}\;\mathrm{of}\;\mathrm{group}1\;\mathrm{or}\;2}{\%\;\mathrm{cumulative}\;\mathrm{mortality}\;\mathrm{of}\;\mathrm{group}\;3}\right)\times 100\%.$$

Liver samples from moribund or freshly dead fish, and five representative surviving fish from each group were collected at the end of experiment at 21 days post-challenge and placed in RNA later (Sigma) at −20 °C for viral load determination. RNA samples were isolated using Trizol following the manufacturer’s protocol (Invitrogen). The quality and quantity of RNA samples were measured with a Nanodrop ND-1000 Spectrophotometer (Thermo Scientific). TiLV viral load was determined by RT-qPCR, by amplifying 137 bp of TiLV segment 9 using specific Seg9-TaqMan-probe (5′-6-FAM-TGC CGC CGC AGC ACA AGC TCC A-BHQ-1-3′), primers Seg9-TaqMan-F (5′-CTAGAC AAT GTT TTC GAT CCA G-3′) and Seg9-TaqMan-R (5′-TTC TGT GTC AGT AAT CTT GAC AG-3′) as described by Taengphu et al. (2021). House-keeping gene *elongation factor-1α (EF1α*) was used as an internal control for the RT-qPCR. To quantify TiLV copy number, a standard curve was produced using ten-fold dilution of plasmid pSeg9-351 containing 351 bp of TiLV segment 9 open reading frame [29] (Figure S1).

### 2.7. Statistical Analysis

GraphPad Prism 6 (GraphPad Software, San Diego, CA, USA) was used to create graphs. The differences on OD_450_ readings representing TiLV-IgM levels were compared with statistically valid cut-off values representing the upper prediction limit using Student *t*-distribution. Cut-off values were calculated using the structures described in Table 2, based on the number of negative control samples and the confidence level of 95% [33]. Kaplan–Meier curves were plotted for cumulative survival rates and the log-rank test was used to compare the differences in survival between groups for the passive immunization experiment.

## 3. Results

### 3.1. Measurement of Systemic Anti-TiLV IgM Levels by ELISA

**Figure 2:**
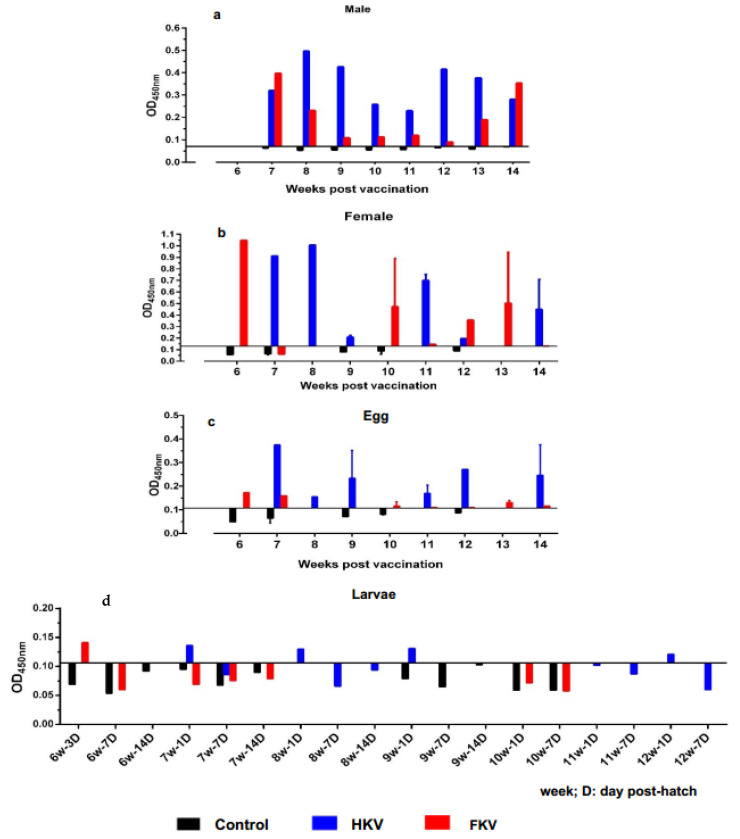
TiLV-specific IgM levels (OD_450_) from 6 to 14 weeks post primary vaccination in (**a**) vaccinated male broodstock (diluted 1:1024, *n* = 1 per treatment weekly), (**b**) vaccinated female broodstock (diluted 1:1024, *n* = 1–2 per treatment weekly), (**c**) egg supernatant (diluted 1:8, *n* = 1–2 per treatment weekly) and (**d**) larval supernatant (diluted 1:2, *n* = 1 per treatment at different sampling time points). The OD_450_ values were compared with significantly statistical cut-off values. HKV, FKV, and Control mean that the broodstock, eggs or larvae originate from the heat-killed vaccine group, the formalin-killed vaccine group and the control group, respectively.

TiLV-specific IgM antibody (anti-TiLV IgM) levels in ELISA were measured as optical density (OD) values at 450 nm (Figure 2) and compared with statistical cut-off values (Table 2). Overall, both male and female broodstock immunized with either HKV or FKV had OD_450_ values above the cut-off value (0.070 and 0.130, respectively) and higher than that of the control group which were lower than the cut-off value during the period from 6 to 14 wppv. There was one exception, where one female broodstock from the FKV group showed an OD_450_ value below the cut-off value (week 7, Figure 2b). There was wide variation in OD_450_ values between individuals, ranging from 0.230 to 0.497 and 0.089 to 0.398 for male broodstock, and from 0.197 to 1.007 and 0.148 to 1.048 for female broodstock that received HKV and FKV, respectively (Figure 2a,b).

In eggs, TiLV-IgM was detected in fertilized eggs from broodstock immunized with both HKV and FKV over the course of the sampling period, with OD_450_ readings above the statistical cut-off value (0.104) and higher than that of the control group (Figure 2c). For the HKV group, the highest TiLV-IgM was detected in the eggs collected at 7 wppv (OD = 0.375), followed by those at 9, 12 and 14 wppv (OD > 0.2), and the lowest values at 8 and 11 wppv with OD values of 0.155 and 0.17, respectively. The FKV group had OD_450_ values much lower than the HKV group, with values ranging from 0.11 to 0.173.

In tilapia larvae, TiLV-IgM was detected in 1-day-old larvae derived from the batches of eggs of the HKV-vaccinated female broodstock at 7, 8, 9 and 12 wppv with OD_450_ values ranging from 0.121 to 0.136 (Figure 2d). On the other hand, TiLV-IgM was detected only in 3-day old larvae derived from the batch of egg of FKV-vaccinated female broodstock at 6 wppv at OD_450_ of 0.141 (Figure 2d). All OD_450_ readings of larvae samples from the control group were below the cut-off value (0.106).

### 3.2. Passive Immunization

**Figure 3:**
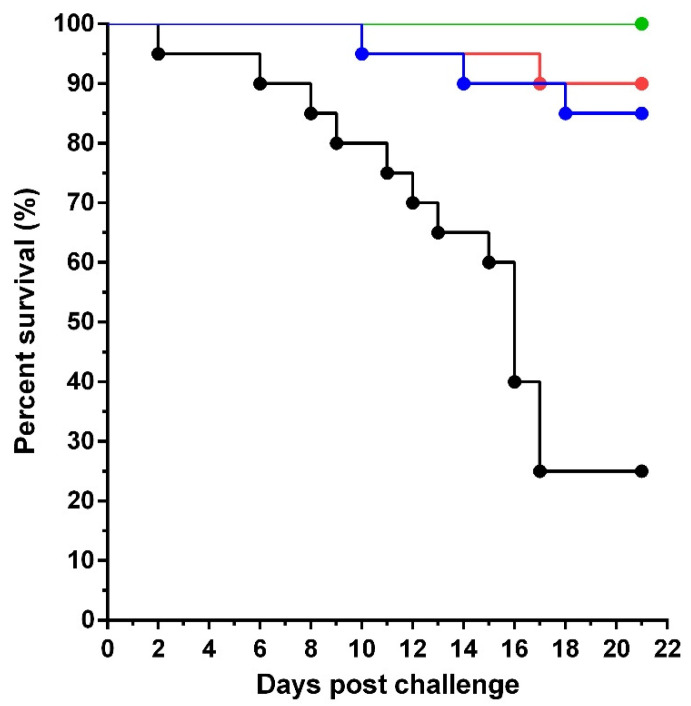
Average percent survival of Nile tilapia juveniles passively immunized with pooled sera from female broodstock by intramuscular injection (IM) and then challenged with TiLV TH-2018 at 9 × 10^5^ TCID_50_ per fish. The differences were statically significant between group 1, 2, 4 and group 3 (*n* = 20 per group, Log Rank test: *p* < 0.0001). HKV, FKV, and control mean broodstock fish were immunized with heat-killed vaccine, formalin-killed vaccine and L15 medium, respectively. The L15 group is negative control group treated with L15 medium without virus (*n* = 20).

After infection with TiLV, the percent survival of the fish receiving sera from HKV and FKV-vaccinated female broodstock (groups 1 and 2) was 85% and 90%, respectively. Conversely, the survival percentage in the group receiving sera from unvaccinated female broodstock (group 3) was only 25%. The differences between groups were statistically significant using a log-rank test (*p <* 0.0001). No mortality was recorded in the negative control (group 4). For the groups vaccinated with HKV sera and FKV sera, an average RPS value of 80% and 86.7%, respectively, was observed (Figure 3).

In all challenge groups, especially in group 3, moribund or dead fish showed a variety of abnormal behaviors and gross lesions. Fish showed loss of appetite, stopped eating, gathered at the corners of the tank and some fish showed erratic swimming. Gross lesions of infected fish showed scale erosion, skin lesions, discoloration. Internally post-mortem changes included gill pallor, liver pallor and ascitic fluid (Figure S2).

Most dead fish in group 3 were TiLV positive by RT-qPCR. The viral load reached a peak at 6 days post challenge (dpc) with a value of 1.4 × 10^6^/µg for RNA template detected, which gradually declined until 17 dpc with a value of 2.5 × 10^1^/µg RNA template recorded (Table S1). There were only two and three dead fish in groups 1 and 2, respectively. However, only one freshly dead fish from each group was found positive for TiLV by RT-qPCR. Viral load was undetectable in surviving fish collected at the end of experiment (21 dpc) for all groups.

## 4. Discussion

In our previous study, vaccination of tilapia juveniles with HKV and FKV resulted in a significant increase in systemic TiLV-specific IgM and high level of protection against TiLV challenge (RPS = 71.3% to 79.6%) [20]. However, the persistence of a specific antibody was not evaluated. In the current study, we used the same vaccination protocol for the tilapia broodstock, using double doses of antigen (3.6 × 10^6^ TCID_50_ per fish compared to 1.8 × 10^6^ TCID_50_ per fish in our previous study) for both primary immunization and the booster vaccination. Relatively high levels of TiLV-IgM were detected from 6 to 14-wppv, suggesting that both HKV and FKV elicited relatively long persistence (98 days) of TiLV-IgM in vaccinated broodstock. This finding is consistent with a previous observation in tilapia juveniles challenged with TiLV, where a specific antibody response was maintained for 6 to 16 weeks post infection [34].

Although the protective efficacy of several TiLV vaccines has been reported recently, the specific role of anti-TiLV antibody against TiLV challenge is still unclear, since several studies reported that TiLV vaccines can stimulate both humoral immunity and cell-mediated immunity [18,19,20]. In this study, the high survival of passive immunized tilapia (85–90%) after receiving sera from the vaccinated broodstock (both HKV and FKV), suggests that humoral immunity plays an important role in protecting against TiLV infection through anti-TiLV antibodies. The reduction in TiLV load during the course of infection, which decreased to undetectable levels in surviving fish by the end of the experiment, reinforces a putative role of protective antibodies in virus clearance. Theoretically, these antibodies could be capable of removing TiLV from the body of the fish by various mechanisms such as neutralization, phagocytosis, antibody-dependent cellular cytotoxicity and complement-mediated lysis of infected cells [35]. Several studies have shown that passive immunization can protect fish from viral infection. For example, intraperitoneal injection of plasma obtained from Pacific herring (*Clupea pallasii*) recovering from a viral hemorrhagic septicemia virus (VHSV) showed that neutralizing antibodies produced against VHSV after infection could protect fish from this virus [36]. Since tilapia broodstock are usually kept in the hatchery for 3 to 5 years [37], vaccination would be an effective strategy to prevent TiLV infection in the broodstock, minimizing economic loss and maintaining good health of the broodstock during the breeding period.

Evidence was provided in the current study that maternal antibodies from TiLV-vaccinated tilapia broodstock are transferred to their offspring. Interestingly, these antibodies were found to be protective during passive immunization in tilapia juveniles challenged with the virus. This suggests that anti-TiLV antibodies may not only help to reduce the risk of infection in broodstock but may also reduce the risk of vertical TiLV transmission. Several studies reported that vaccination of broodstock is an effective strategy to enhance the maternal transfer of immunity from mother to offspring and reduce the risk of vertical transmission of the pathogen. For example, tilapia broodstock vaccinated with inactivated vaccines against *Streptococcus agalactiae* and *Aeromonas hydrophila* were able to induce passive transfer of specific antibodies to eggs and larvae, thus improving the quality and survivability of the offspring [28,38,39]. A bivalent inactivated vaccine against NNV and grouper iridovirus (GIV) administered to grouper (*Epinephelus tukula*) prior to spawning induced neutralizing antibodies against both NNV and GIV [40]. These antibodies were vertically transferred to the eggs and reduced the risk of vertical infection. In another study in grouper (*E. tukula*), antibodies against NNV persisted for up to 17 months following vaccination with an NNV-inactivated vaccine. Five months after vaccination, NNV was no longer detectable in the eggs of the vaccinated group, but was detected in the eggs of the unvaccinated group [27].

Higher levels of TiLV-IgM were found in the fertilized eggs of the group vaccinated with HKV than in those of the fish vaccinated with FKV, suggesting that HKV is more promising for successful maternal vaccination. However, TiLV-IgM transfer only persisted for 1 to 3-days post-hatch and was undetectable by 7 and 14-day post hatch. Because TiLV challenge was unsuccessful at the larval stage of tilapia, we did not evaluate passive antibody protection in the offspring. However, these findings suggest that maternal antibody transfer in larvae does not last long and may be insufficient to protect offspring after 1–3 days post-hatch. This result is in agreement with the results observed in grouper vaccinated against NNV, where NNV-specific antibodies were found to gradually decrease within 48 h after hatching [27]. Such short persistence can be explained by the gradual decline in IgM during yolk-sac absorption observed in tilapia [41], and other fish such as European sea bass (*Dicentrarchus labrax*) [42] and Atlantic salmon (*Salmo salar* L.) [43]. Therefore, in addition to vaccination, biosecurity measures remain essential to prevent the introduction of pathogens into tilapia hatcheries, especially during seed production.

## 5. Conclusions

This study has shown that vaccination of tilapia broodstock with HKV and FKV elicits a protective antibody response against TiLV, and that these antibodies can be transferred to the fertilized eggs and larvae to induce maternal immunity. HKV appears to have greater potential than FKV for maternal transmission of antibodies. However, protective antibodies had a short persistence in the larvae leaving a gap between maternal immunity and immunocompetence. Further vaccination is therefore likely to be needed to protect fish from TiLV infection during this gap as well as later stages of development.

## Figures and Tables

**Table 1:** Batches of fertilized eggs and larvae were collected at different time points post-primary vaccination.

	Time	6 Wppv	7 Wppv	8 Wppv	9 Wppv	10 Wppv	11 Wppv	12 Wppv	13 Wppv	14 Wppv
Treatment										
Control		2 FE 1 L (3D-7D-14D)	2 FE 1 L (1D-7D-14D)		1 FE 1L (1D-7D-14D)	2 FE 1 L (1D-7D)		1 FE		
HKV			1 FE 1 L (1D-7D)	1 FE 1 L (1D-7D-14D)	2 FE 1 L (1D)		2 FE 1 L (1D-7D)	1 FE 1 L (1D-7D)		2 FE
FKV		1 FE 1 L (3D-7D)	1 FE 1 L (1D-7D-14D)			2 FE 1 L (1D-7D)	1 FE	1 FE	2 FE	1 FE

Wppv, week post-primary vaccination; HKV: heat-killed vaccine; FKV: formalin-killed vaccine; FE, batch of fertilized eggs (50 fertilized eggs); L, batch of larvae with different day old; D, day post-hatch.

**Table 2:** Structures for calculation of ELISA cut-off values [33].

Samples	Structures	Cut-Off Values
Female broodstock sera	Mean OD_450_ + 2.077 × SD	0.070
Male broodstock sera	Mean OD_450_ + 2.01 × SD	0.130
Egg supernatant	Mean OD_450_ + 2.01 × SD	0.104
Larval supernatant	Mean OD_450_ + 1.923 × SD	0.106

Standard deviation multipliers (f) were derived from critical values for a one-tailed *t-*distribution with a confidence level of 95% [33], where f = 2.077, 2.01, 2.01 and 1.923 for female broodstock, male broodstock, egg and larval supernatant, respectively.

## Data Availability

The data that support the findings of this study are available on request.

## Supplementary Materials

The following supporting information can be downloaded at: https://www.mdpi.com/article/10.3390/vaccines10020167/s1. Figure S1. Standard curve for viral load calculation; Figure S2. (a) Gross lesions of infected fish showed scale erosion, skin lesions, discoloration. (b) Internal postmortem changes including gill pallor, liver pallor (arrow) and ascitic fluid (head arrow). Sample was taken on 6 dpc from group 3. Scale bar = 1 cm; Table S1. TiLV copy number measured by RT-qPCR targeting RNA segment 9.
