# Supplementary material for: Immunization of Nile Tilapia (Oreochromis niloticus) Broodstock with Tilapia Lake Virus (TiLV) Inactivated Vaccines Elicits Protective Antibody and Passive Maternal Antibody Transfer
Source: Vaccines (Basel). 2022 Jan 21;10(2):167. doi: 10.3390/vaccines10020167 (PMC8879158; doi:10.3390/vaccines10020167)
Supplement: Supplementary file 1 [file vaccines-10-00167-s001.zip › vaccines-1556977-supplementary.pdf]

vaccines

Supplement data

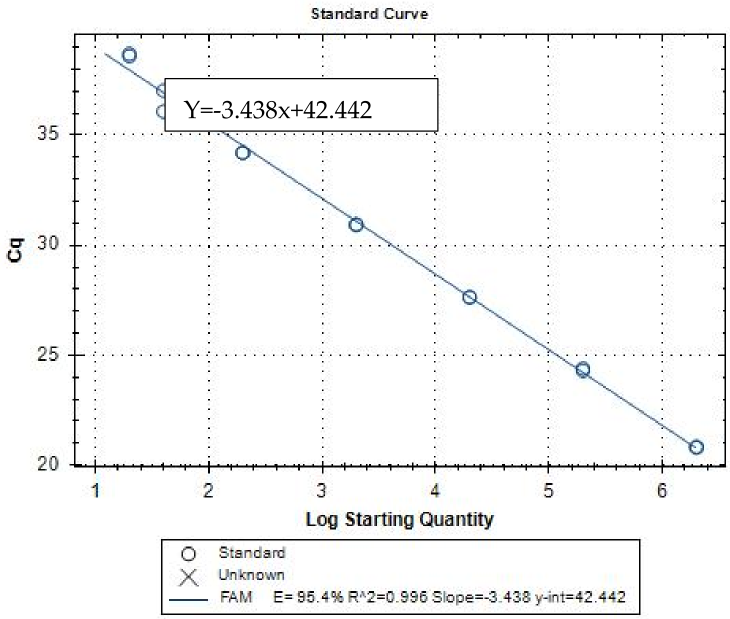

Figure S1. Standard curve for viral load calculation.

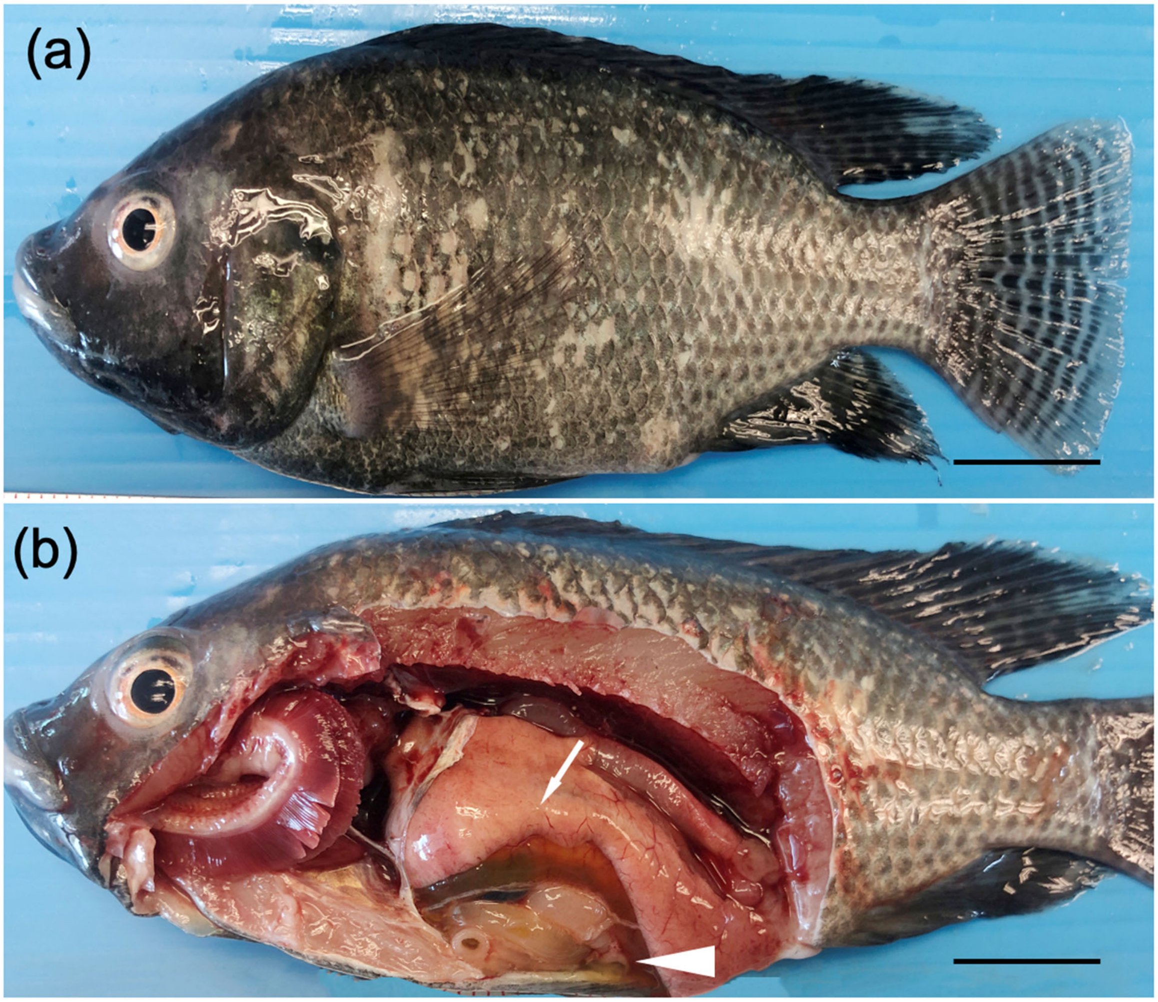

Figure S2. (a) Gross lesions of infected fish showed scale erosion, skin lesions, discoloration. (b) Internal postmortem changes including gill pallor, liver pallor (arrow) and ascitic fluid (head arrow). Sample was taken on 6 dpc from group 3. Scale bar = 1 cm.

Table S1. TiLV copy number measured by RT-qPCR targeting RNA segment 9.

Fish No	Group	Time of death	C_q for TiLV segment 9	C_q for *EF1α*	Viral copy number/ µg RNA template
1	3	Day 2	31.54	19.95	7.4×10^3
2	3	Day 6	22.7	19.52	1.4×10^6
3	3	Day 8	37.27	20.16	1.6×10^2
4	3	Day 9	ND	19.52	0
5	3	Day 11	38.37	19.62	7.5×10
6	3	Day 12	36.15	20.76	3.4×10^2
7	3	Day 16	36.99	21.15	1.8×10^2
8	3	Day 16	34.22	21.95	1.2×10^3
9	3	Day 16	ND	19.43	0
10	3	Day 16	36.74	21.01	2.3×10^2
11	3	Day 17	39.93	19.41	2.5×10
12	1	Day 10	ND	19.81	0
13	2	Day 10	ND	20.11	0
14	1	Day 21	ND	21.01	0
15	1	Day 21	ND	20.91	0
16	1	Day 21	ND	21.04	0

17	1	Day 21	ND	21.21	0
18	1	Day 21	ND	20.90	0
19	2	Day 21	ND	21.79	0
20	2	Day 21	ND	22.40	0
21	2	Day 21	ND	20.24	0
22	2	Day 21	ND	21.58	0
23	2	Day 21	ND	21.25	0
24	3	Day 21	ND	22.44	0
25	3	Day 21	ND	20.37	0
26	3	Day 21	ND	19.06	0
27	3	Day 21	ND	19.59	0
28	3	Day 21	ND	22.31	0

ND: not detectable; C_q: quantification cycle; *EF1α*: *elongation factor-1α* gene.
